# N-Terminally Glutamate-Substituted Analogue of Gramicidin A as Protonophore and Selective Mitochondrial Uncoupler

**DOI:** 10.1371/journal.pone.0041919

**Published:** 2012-07-24

**Authors:** Alexandra I. Sorochkina, Egor Y. Plotnikov, Tatyana I. Rokitskaya, Sergei I. Kovalchuk, Elena A. Kotova, Sergei V. Sychev, Dmitry B. Zorov, Yuri N. Antonenko

**Affiliations:** 1 Belozersky Institute of Physico-Chemical Biology, Lomonosov Moscow State University, Moscow, Russia; 2 Shemyakin and Ovchinnikov Institute of Bioorganic Chemistry, Russian Academy of Sciences, Moscow, Russia; Université Joseph Fourier, France

## Abstract

Limited uncoupling of oxidative phosphorylation could be beneficial for cells by preventing excessive generation of reactive oxygen species. Typical uncouplers are weak organic acids capable of permeating across membranes with a narrow gap between efficacy and toxicity. Aimed at designing a nontoxic uncoupler, the protonatable amino acid residue Glu was substituted for Val at the N-terminus of the pentadecapeptide gramicidin A (gA). The modified peptide [Glu1]gA exhibited high uncoupling activity in isolated mitochondria, in particular, abolishing membrane potential at the inner mitochondrial membrane with the same or even larger efficacy as gA. With mitochondria in cell culture, the depolarizing activity of [Glu1]gA was observed at concentrations by an order of magnitude lower than those of gA. On the contrary, [Glu1]gA was much less potent in forming proton channels in planar lipid bilayers than gA. Remarkably, at uncoupling concentrations, [Glu1]gA did not alter cell morphology and was nontoxic in MTT test, in contrast to gA showing high toxicity. The difference in the behavior of [Glu1]gA and gA in natural and artificial membranes could be ascribed to increased capability of [Glu1]gA to permeate through membranes and/or redistribute between different membranes. Based on the protective role of mild uncoupling, [Glu1]gA and some other proton-conducting gA analogues may be considered as prototypes of prospective therapeutic agents.

## Introduction

Proton fluxes across membranes are of crucial importance for cell functioning. The mostly studied are active fluxes through proton pumps of electron transfer chains providing proton motive force as an energetic intermediate between oxidation and ATP synthesis, in other words, underlying the energetic coupling of electron transfer and phosphorylation in mitochondria, chloroplasts and bacteria [1]. Of the key importance for cellular physiology is also the functioning of proton pumps in endosomes resulting in acidification of their interior which is a prerequisite of their maturation and intracellular traffic. In plasma membranes of eukaryotic cells, proton pumps also play a significant role, e.g. providing intragastric acidification. Another type of passive transmembrane proton fluxes is found in proton channels of plasma membranes which determine such vital processes as immune responses of certain kinds of blood cells [2].

A breakthrough in uncovering the mechanism of oxidative phosphorylation in mitochondria was promoted by the early observations that some aromatic weak acids (e.g., 2,4-dinitrophenol) are able to selectively transfer protons across artificial and natural membranes thereby leading to uncoupling of electron transfer and phosphorylation. The capability of uncoupling was also found to be characteristic of a new class of membrane proteins, the so-called uncoupling proteins (UCP), which appeared to cause a reduction of the mitochondrial membrane potential in the presence of fatty acids [3], [4]. To this end, it is of importance that a high magnitude of the mitochondrial membrane potential could be harmful for cells through generation of excess of reactive oxygen species (ROS) provoking a series of pathological processes in tissues [3], [5]–[7]. By contrast, a moderate decrease in membrane potential was shown to protect cells from oxidative damage [8]–[11]. Thus, protonophores represent potential drugs [12]. Actually in 1930-s 2,4-dinitrophenol was used as a drug against obesity. However, later it was prohibited due to high toxicity ([13] and refs. therein).

Cytotoxicity of uncouplers is generally attributed to an excessive increase in proton conductivity of the inner mitochondrial membrane [14], although ionic balance across other cellular membranes can be also changed by uncouplers [15]. Reduction of the toxicity might be associated with voltage dependence of their action, e.g. a drop in activity upon partial depolarization of the inner mitochondrial membrane, which may prevent an uncontrolled decrease in ATP synthesis and cell death. It is well-known that proteinaceous channels exhibit gating and vivid voltage dependence. Bearing in mind a large body of evidence on proton conductivity of certain proteinaceous channels, it seems reasonable to consider peptide protonophores as candidates for low-toxic uncouplers. The ionic channel formed by the pentadecapeptide gramicidin A (gA) is known to effectively conduct protons [16]–[22]. Unfortunately, it cannot be used as a protonophoric drug in mammalian cells because of high toxicity [23] associated with its high conductivity for all monovalent cations, in particular, potassium and sodium [24].

It is generally accepted that in bilayer lipid membranes gA acquires a β^6.3^–helical conformation and head-to-head association of two gA molecules via six hydrogen bonds leads to formation of a transmembrane channel. In membranes of certain lipid composition, gA channels were supposed to have an alternative structure of a β^5.6^-double-helix [25], [26]. N-terminal modifications of gA were shown to affect substantially its channel properties, leading even to complete blockage of channel formation [27]–[34]. Noteworthy, almost complete loss of potassium conductivity in case of desformylgramicidin was combined with much less reduction of proton conductivity with respect to parent gA [35], [36], so that the desformylated peptide was able to uncouple oxidative phosphorylation [37]. However, its toxicity was not evaluated. The present study dealt with a series of gA analogues that were N-terminally modified with protonatable amino acid residues – glutamic acid and lysine. The analogue [Glu1]gA having a glutamic acid residue in position 1 appeared to be even more potent in lowering the mitochondrial membrane potential than the parent gA peptide. The protonophoric activity of [Glu1]gA was studied in different systems: mammalian cells, isolated mitochondria, liposomes and planar lipid bilayers. Remarkably, [Glu1]gA in micromolar concentrations decreased the mitochondrial membrane potential in rat kidney cell culture, being nontoxic to the cells under these conditions.

## Results

**Figure 1:**
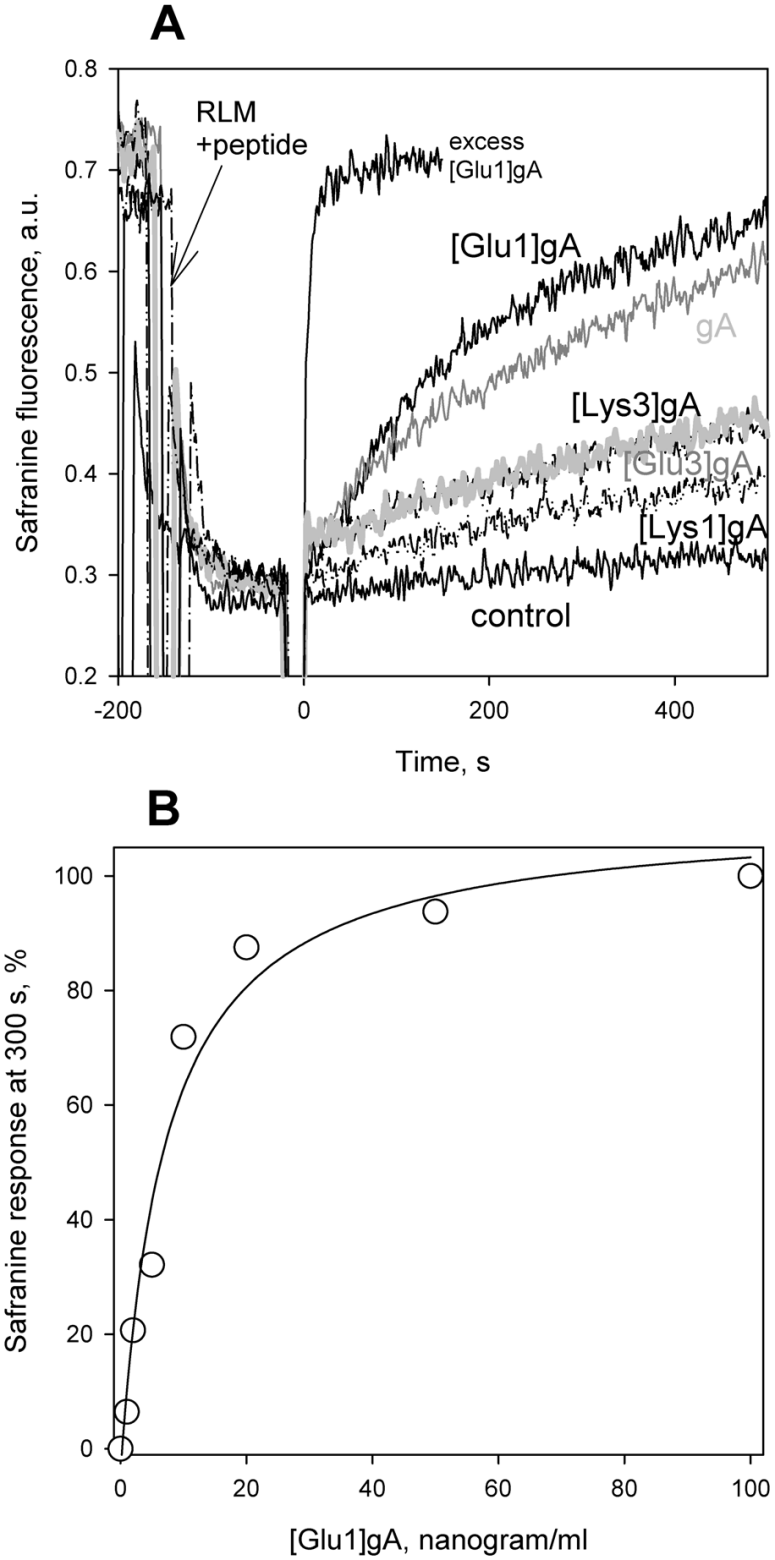
Effect of different peptides on the membrane potential of rat liver mitochondria measured by safranine O. Panel **A**. Shown are traces of fluorescence in the medium described in “Materials and Methods.” In all traces, 5 mM of succinate and 1 µM of rotenone were supplemented about 150 s before the addition of a peptide at t = 0 s. Control, no other additions. gA, [Glu1]gA, [Glu3]gA, [Lys1]gA, and [Lys3]gA show traces after the addition of 5 nM (i.e. about 10 nanogram/ml) of a corresponding peptide at t = 0 s. Trace “excess [Glu1]gA” was measured with 1 µg/ml peptide. Panel **B.** Dose dependence of the effect of [Glu1]gA on mitochondrial membrane potential.

Protonophores are known to collapse the difference of electric potentials across the inner mitochondrial membrane [38]–[43] that causes uncoupling of oxidation and phosphorylation [44]–[46]. To assess the effect of gramicidin derivatives on the mitochondrial membrane potential, we assayed the latter with the potential-dependent dye safranine O. The fluorescence of this dye (at a high concentration) is known to drop with increasing the mitochondrial membrane potential and rise upon the addition of protonophores [42], [47]. Figure 1A displays time courses of fluorescence intensity of safranine O added to a suspension of isolated rat liver mitochondria which were energized by the addition of succinate in combination with rotenone about 150 s before being supplemented with low concentration of peptides (5 nM or about 10 nanogram/ml) at t = 0 s. It can be seen that the glutamate-substituted gA analogue [Glu1]gA and the parent gA were most efficient in decreasing the mitochondrial membrane potential. Figure 1B shows dose dependence of the effect of [Glu1]gA. The concentration dependence of the relative drop in the membrane potential (measured at 300 s after the peptide addition) showed the following series of the uncoupling potency of the analogs: [Glu1]gA > = gA > [Glu3]gA > [Lys3]gA > [Lys1]gA. Notably, the concentrations of the cationic gA analogues [Lys3]gA and [Lys1]gA required to decrease the membrane potential were similar to those of the mitochondrial presequence and other cationic peptides [48]. At concentrations higher than 100 nM (200 nanogram/ml), [Glu1]gA stimulated mitochondrial respiration up to the level of fully uncoupled respiration (data not shown).

**Figure 2:**
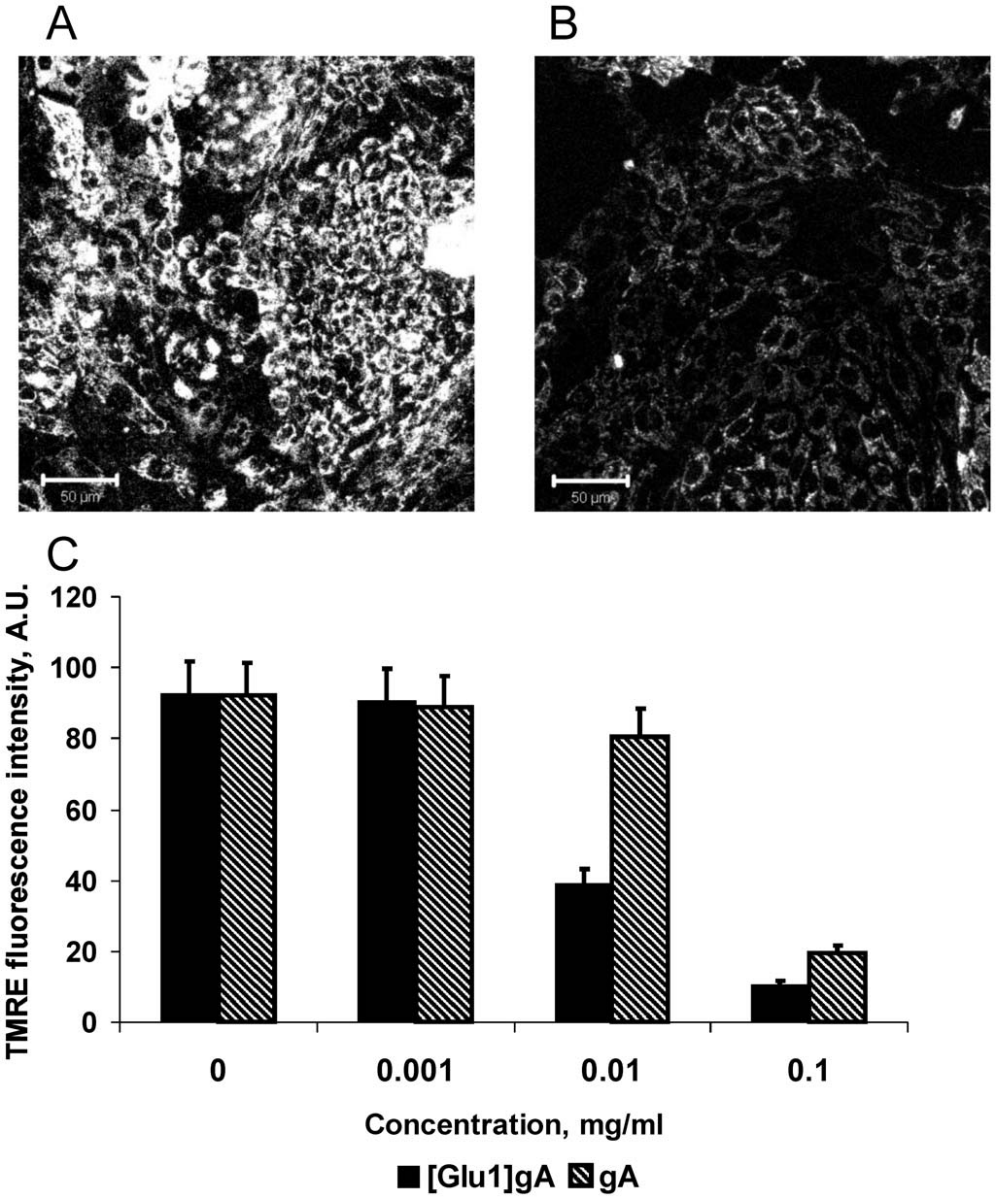
Effect of [Glu1]gA on mitochondrial membrane potential in renal cells. Confocal images of cultured renal cells loaded with TMRE in control (A) and after treatment with 0.01 mg/ml [Glu1]gA (B). Diagram (C) presents the mean intensity of TMRE fluorescence through 10 confocal images for each [Glu1]gA (solid bars) or gA (hatched bars) concentration.

**Figure 3:**
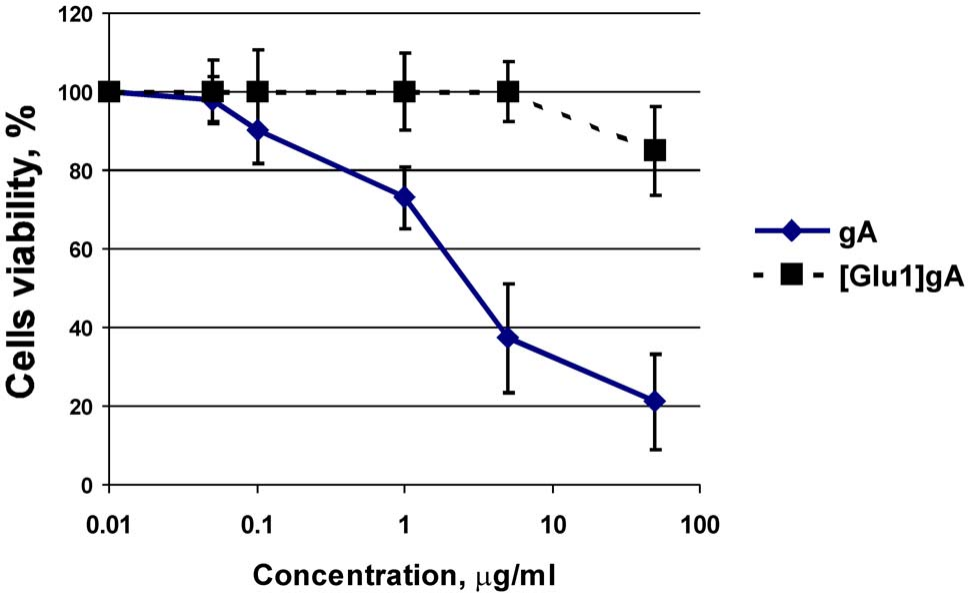
Viability of cultured renal cells according to the MTT test after 24 h treatment with appropriate concentration of [Glu1]gA or gA in culture medium without serum. Experiments were performed 2 times in which 10 wells per each concentration were analyzed.

In subsequent series of experiments, the uncoupling action of [Glu1]gA was tested in rat kidney cell culture loaded with the potential sensitive dye tetramethylrhodamine ethyl ester (TMRE). As seen from micrographs (Fig. 2), 10 µg/ml (i.e. about 5 µM) of [Glu1]gA caused more than a two-fold decrease in the mitochondrial membrane potential without noticeable changes in the cell shape. By contrast, the parent gA depolarized mitochondria in cells at substantially higher concentrations starting from 0.1 mg/ml (Fig. 2C). Practically no change in cell viability was detected at this concentration of [Glu1]gA by the standard MTT test showing the viable cell number (Fig. 3). In similar experiments gA showed marked cytotoxicity for renal tubular cells, compared to [Glu1]gA (Fig. 3). In particular, treatment with 50 µg/ml gA caused about 80% cell death, while [Glu1]gA provoked only about 15% cell death. Concentrations below 5 µg/ml were nontoxic in case of [Glu1]gA, whereas gA possessed certain toxicity up to 0.1 µg/ml. Therefore, in contrast to the parent gA, [Glu1]gA proved to be an effective and nontoxic uncoupler at this concentration. The increased effective concentrations of the [Glu1]gA in cell culture with respect to those with isolated mitochondria (Fig. 1) could be attributed to poor peptide permeation through plasma membrane.

**Figure 4:**
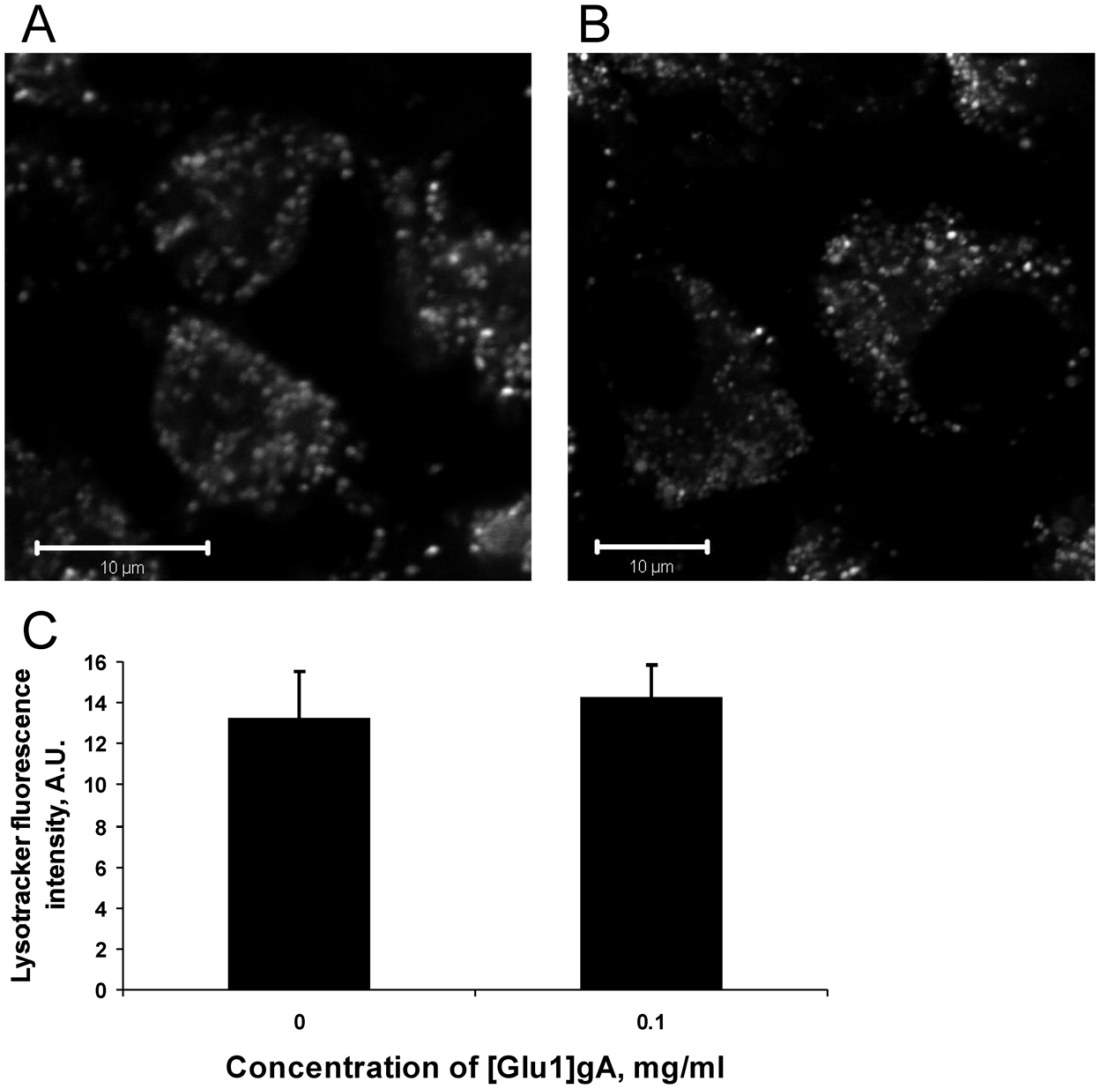
Micrographs of rat kidney cell culture loaded with Lysotracker Green in the absence (A), and in the presence of 0.1 mg/ml (B) of [Glu1]gA.

In further experiments, we tested the interaction of [Glu1]gA with lysosomes of rat kidney cells by using the dye Lysotracker Green known to accumulate in highly acidic interior of lysosomes. No change both in lysosome integrity and pH gradient across lysosomal membranes (in terms of Lysotracker fluorescence intensity) was observed (Fig. 4) even after 0.1 mg/ml [Glu1]gA treatment that led to maximal depolarization of mitochondria. Thus, [Glu1]gA appeared to interact specifically with mitochondrial membranes leading to their partial depolarization.

**Figure 5:**
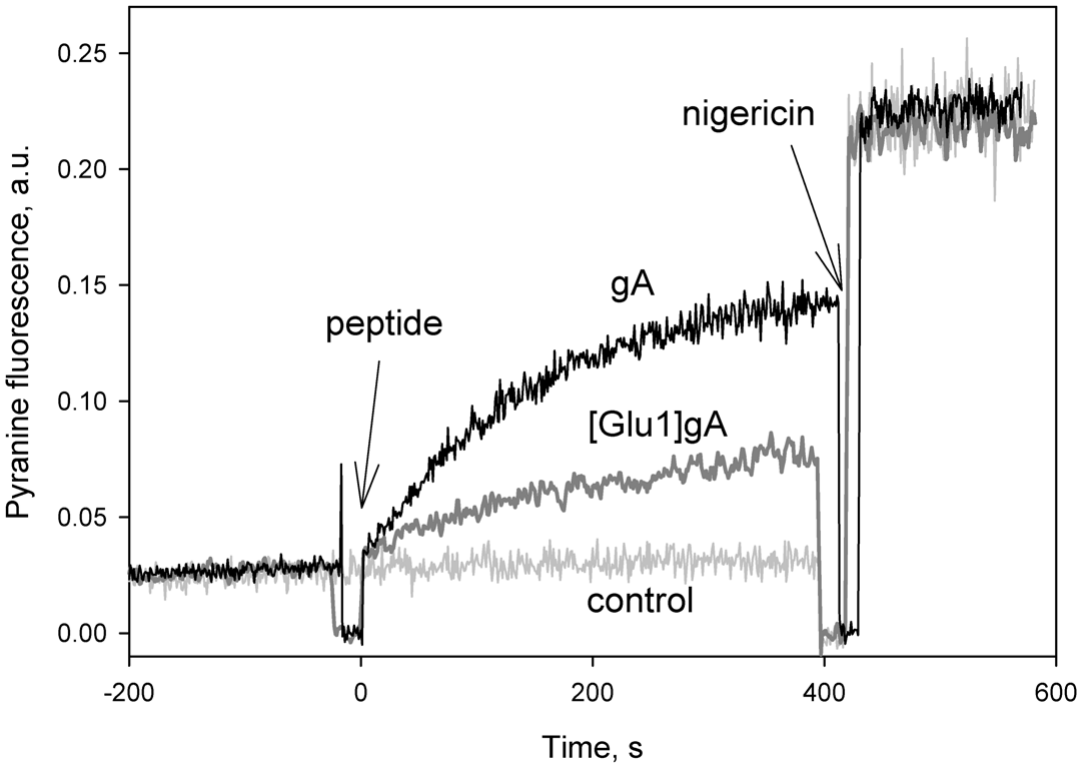
Dissipation of the pH gradient on membranes of pyranine-loaded liposomes by gA and [Glu1]gA (both 2 µg/ml). Inner liposomal pH was estimated from pyranine fluorescence intensity measured at 505 nm upon excitation at 455 nm. Nigericin (1 µM) was added at 400–420 s to equilibrate the pH. Control, a record without peptides.

To verify the suggestion that the collapse of both mitochondrial and bacterial membrane potential by the glutamate-substituted gA analogues is associated with protonophoric activity of the peptides, we measured their effect on proton fluxes across model membranes via recording the fluorescence of the pH-sensitive dye pyranine [49]. With the pH gradient created across liposomal membranes (pH_in_ = 8, pH_out_ = 6), [Glu1]gA brought about an enhancement of fluorescence of liposome-entrapped pyranine, though slower than that provoked by the parent peptide gA (Fig. 5).

**Figure 6:**
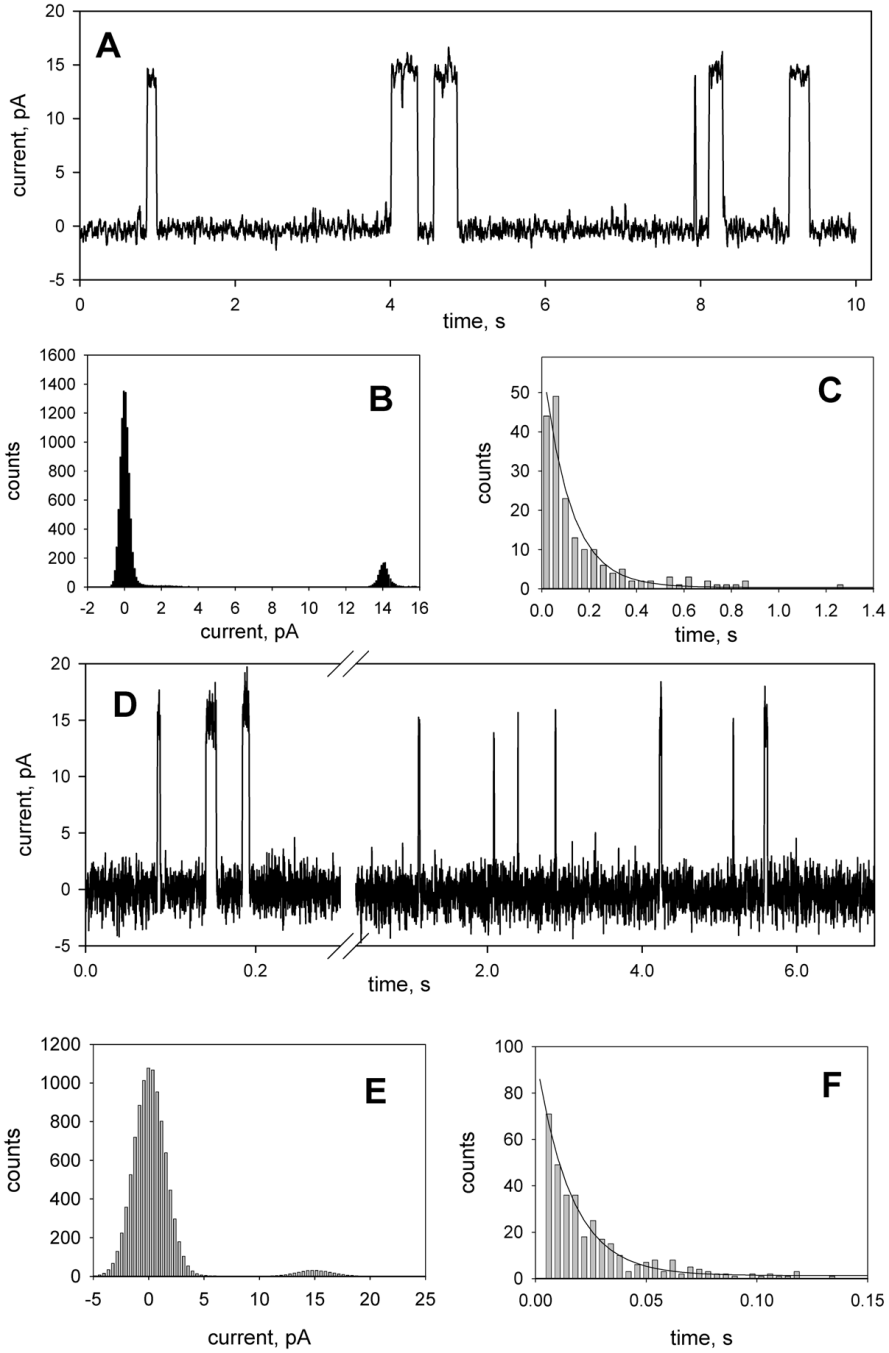
Single-channel recordings (A, D) and the corresponding current histograms (B, E) of gramicidin A (picogram/ml, A, B, and C) and [Glu1]gA (2 nanogram/ml, D, E, and F) at a voltage of 100 mV applied to the DPhPC/decane membrane. The solution was 100 mM HCl. (**C**, **F**) Open state duration histograms fitted by a single exponential with a time constant of 116 ms (**C**) and 16 ms (**F**). The records were filtered at 100 Hz (**A**, **B**, and **C**) or 1000 Hz (**D**, **E**, and **F**).

Proton channels formed by gA can be observed by measuring electric current across a planar bilayer lipid membrane (BLM), if the experiments are performed in the acidic pH range [19], [20], [26], [50]. Figure 6 presents typical recordings of fluctuations of transmembrane current induced by gA and [Glu1]gA (panels A and D of Fig. 6) which were obtained under the same conditions. The single-channel conductance of [Glu1]gA (150 pS) was close to that of gA (140 pS) as judged from the current histograms (panels B and E), while the single-channel lifetime of the analogue (about 16 ms as estimated from the exponential fit to open channel duration histogram, panel F of Fig. 6) was much shorter than that of gA (116 ms, panel C). Besides, to observe single channels, much higher (approx. by two orders of magnitude) concentrations of [Glu1]gA than those of gA were required.

**Figure 7:**
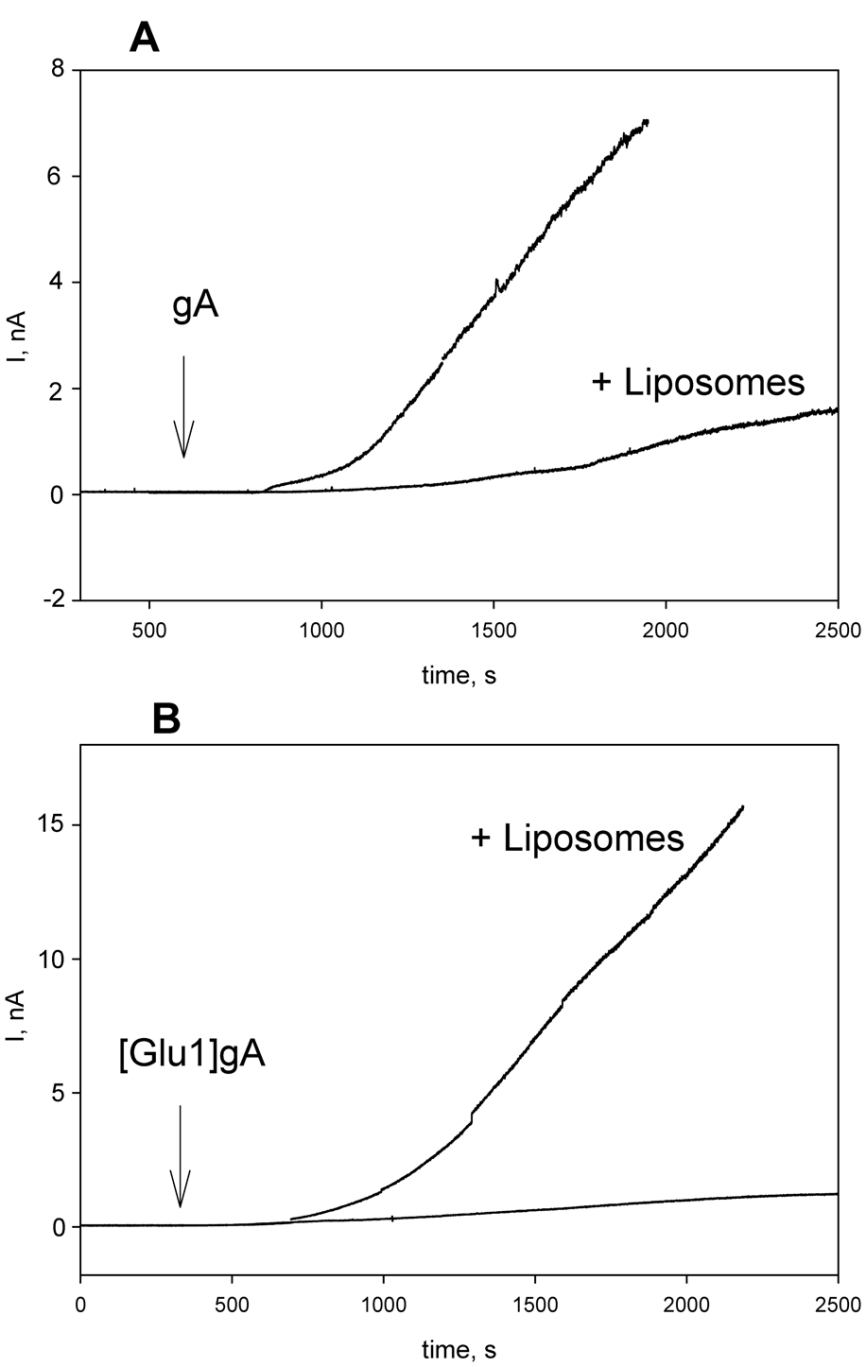
Effect of liposomes (1 µg/ml DPhPC) on the induction of electrical current on planar lipid bilayer by gA (2 nanogram/ml, panel A) and [Glu1]gA (200 nanogram/ml, panel B). The peptides and liposomes were added at one side of the membrane. The solution was 100 mM KCl, 10 mM Tris, 10 mM MES, pH = 7.0, BLM voltage was 50 mV.

The difference in efficacy of gA and [Glu1]gA described here in a variety of systems could be associated with different abilities of the peptides to redistribute between membrane structures and/or their diverse permeability through membranes. To cause a reduction of an electrical potential difference across the inner mitochondrial membrane, peptide molecules must penetrate through the outer membrane in the case of isolated mitochondria, and additionally through the plasma membrane in the case of cells. While encountering with a series of membrane structures, e.g. endosomes, on their way from the plasma to the mitochondrial membrane, peptides may undergo numerous cycles of membrane binding and desorption. To study such a process in a model system, we measured the effect of liposome addition on the time course of the induction of electrical current across a planar BLM by gA and [Glu1]gA. In these experiments we added liposomes and peptides at one side of BLM in order to mimic the addition of the peptides to mitochondria and cells. Figure 7 shows typical recordings after the addition of gA (panel A) and [Glu1]gA (panel B) to BLM in the absence and in the presence of liposomes. The induction of the gA current was somewhat slower in the presence of liposomes. By contrast, in the case of [Glu1]gA the addition of liposomes accelerated many fold the induction of the current. These data can be associated with the ability of [Glu1]gA channels but not of gA channels, after being pre-formed in liposomes, to exchange between membranes and move to planar BLM, thus leading to activation of the BLM current. Interestingly, the channel-forming capacity of [Glu1]gA, when added from one side of the membrane, was much lower than that of gA (i.e. concentrations of about two orders of magnitude higher were required in case of [Glu1]gA to induce the current similar to that of gA), similarly to the difference found for symmetrical additions (shown in Fig. 6). As the channel is supposed to consist of two peptide monomers each spanning one monolayer of the membrane, this observation suggests the step of transmembrane translocation to be not rate-limiting in the induction of the current. However, this result does not necessarily rule out the possibility that the difference in the channel activity in mitochondria and liposomes can result from different permeation abilities of these peptides because permeation implies an additional step of the peptide transfer into the water phase on the opposite side of the membrane.

## Discussion

Controlled uncoupling that prevents undesirable consequences of excessive mitochondrial membrane potential should be one of the major goals of pharmacology. Gramicidin A conventionally used as an uncoupler in isolated mitochondria [51] and chloroplasts [52] could be considered as a candidate for mitochondria uncoupling in tissues. However, gA is characterized by high toxicity [23], in particular, it perturbs ion balance across plasma membrane even at low concentrations [53]–[55]. The gA toxicity also observed here (Fig. 3) is apparently associated with the high potency of the peptide to form channels that selectively conduct monovalent cations, in particular, potassium and sodium. Therefore, to avoid the gA toxicity, one could try to alter its ionic selectivity by modifying the amino acid sequence or attaching certain groups to its N-termini. As shown in our previous work [56], substitutions of lysine for position 1 or 3 at neutral pH when this residue is positively charged, strongly diminished the peptide channel-forming potency which could suppress its toxicity for cells. In the present study we described the uncoupling activity of the glutamate-substituted gA analogue ([Glu1]gA) in cells and isolated mitochondria which was supported by the data on its proton-carrying activity in a series of model systems. Remarkably, having even higher than gA uncoupling activity (Fig. 2C), [Glu1]gA was nontoxic for rat kidney primary cell culture, in contrast to gA (Fig. 3). The difference in toxicity could be associated with the fact that glutamate residues are mostly deprotonated at pH 7 and thus the majority of [Glu1]gA molecules would carry negative charges at their N-termini, which prevents formation of the conducting parthway for monovalent cations through head-to-head association of two monomers. Moreover, even the uncharged form of [Glu1]gA might form a distorted dimer in overall β^6.3^–helical conformation with reduced potassium conductance. This follows from the two-orders of magnitude difference in the concentrations of gA and [Glu1]gA required to induce channel formation in BLM (Fig. 6). Besides, in liposomes, [Glu1]gA was also much less active than gA (Fig. 5).

To reconcile the lower activity of [Glu1]gA as compared to gA in model membranes with the higher activity of the analogue as compared to the parent peptide in isolated mitochondria (which is even more pronounced in cell culture), one should take into account additional membrane barriers encountered by peptide molecules on their way to the target (inner mitochondrial membrane), i.e. the outer membrane of mitochondria and plasma membrane of cells, on one hand, and the absence of such barriers in the case of liposomes and planar bilayers, on the other hand. If one assumes that membrane permeability of [Glu1]gA exceeds that of gA, then the uncoupling potency of [Glu1]gA would be higher than that of gA especially in cells, where the peptide should permeate through at least two membranes before reaching the inner mitochondrial membrane. The fact that the effective concentrations were higher in cells than in mitochondria by three orders of magnitude for [Glu1]gA and to a larger degree for gA proves the permeation through the membrane to be a limiting stage in the uncoupling action of the peptides. This idea is in line with the results of the experiments with peptides preincubated with liposomes (Fig. 7).

The present study has shown that peptides can be used for membrane depolarization along with conventional uncouplers such as FCCP (carbonyl cyanide-p-trifluoromethoxyphenylhydrazone) not only in isolated mitochondria, but also in mammalian cells. Historically, gramicidin was the first antibiotic drug, although its systemic use in clinical practice was prevented by the high toxicity for eukaryotic cells. Comprehensive examination of gA as the first channel with an exactly characterized molecular structure was aimed mostly at elucidating the mechanism of ionic channel operation [57]–[59] and peptide interaction with lipid membrane environment. The studies of gA and its engineered analogues have produced a large body of basic knowledge and, moreover, substantially advanced a variety of experimental approaches. In the present work, we described the newly synthesized [Glu1]gA peptide exhibiting very low toxicity towards eukariotic cells, which is obviously associated with suppressed conductivity of the peptide for potassium and sodium ions. Remarkably, the protonophoric activity of [Glu1]gA resulted in mitochondria uncoupling at the peptide concentrations which appeared to be practically nonpoisonous for mammalian cells. Bearing in mind that partial uncoupling is known to protect mitochondria from oxidative stress and produce a beneficial effect on organisms against obesity, [Glu1]gA can be considered as a prototype of a new class of peptide therapeutic agent.

## Materials and Methods

### Synthesis of gA Analogues

Analogs of gramicidin A, i.e. [Glu1]gA (HCO-EGALAVVVWLWLWLW-NH(CH_2_)_2_OH), [Glu3]gA (HCO-VGELAVVVWLWLWLW-NH(CH_2_)_2_OH), [Lys1]gA (HCO-KGALAVVVWLWLWLW-NH(CH_2_)_2_OH) and [Lys3]gA (HCO-VGKLAVVVWLWLWLW-NH(CH_2_)_2_OH), were prepared by standard solid-phase Nα-Fmoc methodology on Rink amide resin [4(2′,4′-Dimethoxyphenyl-Fmoc-aminomethyl)-phenoxy resin] using the diisopropylcarbodiimide/1-hydroxybenzotriazole coupling system. N-terminal formylation of peptides was conducted in the presence of N-ethyldiisopropylamine using 2-nitrophenyl formate. The peptide resins were treated with trifluoroacetic acid-ethandithiol-water (94:3:3) for 2.5 h. HPLC-purification of the samples gave pure peptides (purity >95%). The fidelity of the peptides was confirmed by MALDI-TOF MS. Gramicidin A (gA) was from Fluka.

### Isolation of Rat Liver Mitochondria

Experiments were performed on outbred white male rats (180–200 g) fed ad libitum. Animal protocols were approved by the Institutional Review Boards. Rat liver mitochondria were isolated by differential centrifugation [60] in a medium containing 250 mM sucrose, 10 mM MOPS, 1 mM EGTA, and bovine serum albumin (0.1 mg/ml), pH 7.4. The final washing was preformed in the medium of the same composition. Protein concentration was determined using bicinchoninic acid as described in [61]. Handling of animals and experimental procedures were conducted in accordance with the international guidelines for animal care and use and were approved by the Institutional Ethics Committee of A.N. Belozersky Institute of Physico-Chemical Biology at Moscow State University.

### Mitochondrial Membrane Potential Measurement

Safranine O (10 µM) was used as a membrane potential probe [42]. Fluorescence intensity at 580 nm (excitation at 520 nm) was measured with a Panorama Fluorat 02 spectrofluorimeter (Lumex, Russia). The medium for measurements contained 300 mM sucrose, 20 mM MOPS, 0.5 mM EDTA, 0.5 mg/ml BSA, pH 7.4. Mitochondrial protein concentration was 0.4 mg/ml.

### Mitochondrial Respiration

Respiration of isolated mitochondria was measured using a standard polarographic technique with a Clark-type oxygen electrode (“Oroboros”, Austria) at 25°C using DATLAB software. The incubation medium contained 300 mM sucrose, 20 mM MOPS, 0.5 mM EDTA, 5.5 mM MgCl_2_, 5 mM KH_2_PO_4_, 0.5 mg/ml BSA, pH 7.4.

### Experiments with Renal Tubular Epithelium Cell Cultures

Kidneys were excised aseptically from 3–7 day old rats, then homogenized and placed in balanced Hanks solution at pH 7.4. After several washes the dispensed tissue was placed in 0.1% collagenase and incubated for 20–30 min at 37°C. Large pieces were removed, and cells were sedimented by gentle centrifugation (50 g) for 3 min. The pellet was resuspended in DMEM/F-12 1:1 containing 10% fetal calf serum (FCS) and seeded in culture plates and glass-bottom dishes. Cells were cultivated in a CO_2_ (5%) incubator for 1–2 days before the experiments. The gA or [Glu1]gA solution was added to the cultured epithelial cells to appropriate concentration and incubated for 10 min at 37°C in DMEM/F-12 medium containing 10 mM Hepes-NaOH. Next, cells were incubated with 100 nM TMRE (Invitrogen, USA) or 1 µM Lysotracker Green (Invitrogen, USA) for 30 min followed by washing in DMEM/F-12. Then renal tubular epithelial cells were imaged with an LSM510 inverted confocal microscope (Carl Zeiss Inc., Jena, Germany). Analysis of fluorochrome incorporation was performed in glass-bottom dishes (MatTek Corp., USA) with excitation at 488 and 543 nm and emission collected at 510–530 and 560–590 nm, for Lysotracker and TMRE, correspondingly. To minimize the contribution of photo-induced mitochondria/cell damage to the relative fluorescence intensities, image analysis was performed on the average of the first four scans only. Images were processed using ImageJ software (NIH, Bethesda, MD, USA).

Cell viability was evaluated by a widely used methyl thiazol tetrazolium (MTT) test. Micromodification of the method was used for 96-well plates for microcultivation [62] Briefly, cells were incubated over 24 hours with appropriate concentration of [Glu1]gA or gA in DMEM/F12 culture medium without serum. After the incubation, cells were washed twice with medium, and the MTT solution was added (2 mg/ml in DMEM/F12) for 60 min at 37°C. Then the dye solution was removed, 50 µl DMSO per well was added and absorbance at 540 nm was measured. Experiments were performed twice in which 10 wells per each concentration were analyzed.

### Proton Transport in Pyranine-loaded Liposomes

The lumenal pH of the liposomes was assayed with pyranine [49] as described in [11] by a slightly modified procedure of [63]. To prepare pyranine-loaded liposomes, lipids (5 mg egg yolk phosphatidylcholine and 1 mg cholesterol) in a chloroform suspension were dried in a round-bottom flask under a stream of nitrogen. The lipids were then resuspended in buffer (100 mM KCl, 20 mM MES, 20 mM MOPS, 20 mM Tricine titrated with KOH to pH 6.0) containing 0.5 mM pyranine. The suspension was vortexed and then freeze-thawed three times. Unilamellar liposomes were prepared by extrusion through 0.1-µm-pore size Nucleopore polycarbonate membranes using an Avanti Mini-Extruder. The unbound pyranine was then removed by passage through a Sephadex G-50 coarse column equilibrated with the same buffer solution. To measure the rate of pH dissipation in liposomes with lumenal pH 6.0, the liposomes were diluted in a solution buffered to pH 8 and supplemented with 10 mM p-xylene-bis-pyridinium bromide to suppress the fluorescence of leaked pyranine. The pH was estimated from the intensities of fluorescence measured at 505 nm upon excitation at 455 nm, as monitored with the Panorama Fluorat 02 spectrofluorimeter. At the end of each recording, 1 µM nigericin was added to dissipate the remaining pH gradient.

### Single Channel Experiments

Planar bilayer lipid membranes (BLM) were formed from a 2% solution of diphytanoylphosphatidylcholine (DPhPC) (Avanti Polar Lipids, Alabaster, AL) in *n*-decane on a hole in a Teflon partition (0.5-mm diameter) separating two compartments of a cell containing aqueous buffer solutions [64].The electric current (I) was recorded under voltage-clamp conditions. Voltage was applied to BLMs with Ag-AgCl electrodes placed directly into the cell. The current measured by means of a patch-clamp amplifier (Warner Instruments, Hamden, CT, model BC-525C) was digitized using an NI-DAQmx (National Instruments, Austin, TX) and analyzed with a personal computer with the use of WinWCP Strathclyde Electrophysiology Software designed by J. Dempster (University of Strathclyde, UK).
